# Supplementary material for: Mechanism of Pressure-Sensitive Adhesion in Nematic Elastomers
Source: Macromolecules. 2023 Aug 10;56(16):6247–55. doi: 10.1021/acs.macromol.3c01038 (PMC10448750; doi:10.1021/acs.macromol.3c01038)
Supplement: Supplementary file 5 — ma3c01038_si_005.pdf [file ma3c01038_si_005.pdf]

Mechanism of pressure sensitive adhesion in nematic elastomers

Hongye Guo, Mohand O. Saed, and Eugene M. Terentjev

Supporting Information

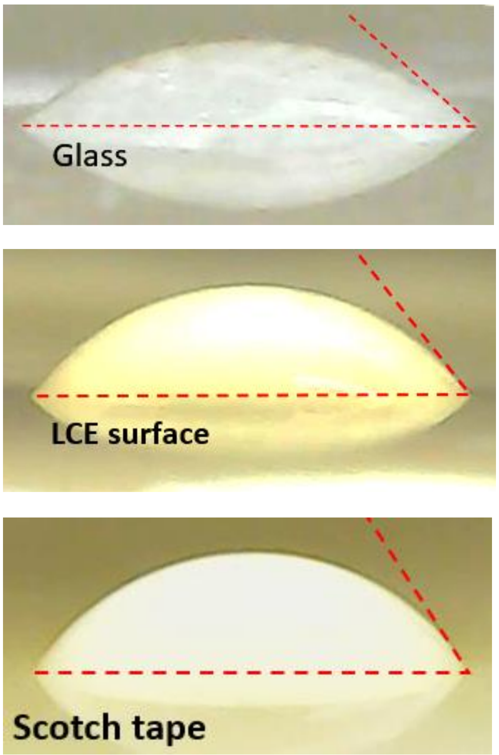

Contact angle of de-ionized water on different surfaces, producing on average:

Glass: $\theta=41^{o}$

LCE: $\theta=52^{o}$

Scotch tape: $\theta=56^{o}$

This illustrates that the “chemical” surface tension is not very different between these surfaces, while our measurements show the large difference of PSA strength.

Supporting Videos:

Video-S1.mp4 – shows the zoomed-in dynamics of debonding in Probe Tack 2 test, with the glass spherical probe lifting from the LCE surface after a small loading. The video collates all three LCE materials, the 5% crosslinked (sample 1.05), the 10% crosslinked (sample 1.1), and the 20% crosslinked (sample 1.2).

Video-S2.mp4 – shows the zoomed-in dynamics of debonding in 90-degrees Peel test, with the PET backing tape with LCE adhesive layer lifting from the flat glass surface after a standard pressure loading. The video collates all three LCE materials, the 5% crosslinked (sample 1.05), the 10% crosslinked (sample 1.1), and the 20% crosslinked (sample 1.2).

Video-S3-5x.mp4 – shows the zoomed-in dynamics of Lap Shear test debonding, viewed from directly above the edge of adhered tape. This video illustrates the 5% crosslinked LCE adhesive layer reaching an instability and then creeping along the surface leaving the residue.

Video-S3-20x.mp4 – shows the zoomed-in dynamics of Lap Shear test debonding, viewed from directly above the edge of adhered tape. This video illustrates the 20% crosslinked LCE adhesive layer reaching an instability and then cleanly debonding from glass leaving the residue.
